# TRAIL delivery by MSC-derived extracellular vesicles is an effective anticancer therapy

**DOI:** 10.1080/20013078.2017.1265291

**Published:** 2017-01-18

**Authors:** ZhengQiang Yuan, Krishna K. Kolluri, Kate H. C. Gowers, Sam M. Janes

**Affiliations:** Lungs for Living Research Centre, UCL Respiratory, Division of Medicine, University College London, London, UK

**Keywords:** EV, cancer, MSC, TRAIL

## Abstract

Extracellular vesicles (EVs) are lipid membrane-enclosed nanoparticles released by cells. They mediate intercellular communication by transferring biological molecules and therefore have potential as innovative drug delivery vehicles. TNF-related apoptosis-inducing ligand (TRAIL) selectively induces apoptosis of cancer cells. Unfortunately, the clinical application of recombinant rTRAIL has been hampered by its low bioavailability and resistance of cancer cells. EV-mediated TRAIL delivery may circumvent these problems. Mesenchymal stromal cells (MSCs) produce EVs and could be a good source for therapeutic EV production. We investigated if TRAIL could be expressed in MSC-derived EVs and examined their cancer cell-killing efficacy. EVs were isolated by ultracentrifugation and were membranous particles of 50–70 nm in diameter. Both MSC- and TRAIL-expressing MSC (MSCT)-derived EVs express CD63, CD9 and CD81, but only MSCT-EVs express surface TRAIL. MSCT-EVs induced apoptosis in 11 cancer cell lines in a dose-dependent manner but showed no cytotoxicity in primary human bronchial epithelial cells. Caspase activity inhibition or TRAIL neutralisation blocked the cytotoxicity of TRAIL-positive EVs. MSCT-EVs induced pronounced apoptosis in TRAIL-resistant cancer cells and this effect could be further enhanced using a CDK9 inhibitor. These data indicate that TRAIL delivery by MSC-derived EVs is an effective anticancer therapy.

## Introduction

Extracellular vesicles (EVs) are cell-released submicron membranous vesicles composed of lipids, proteins and nucleic acids. Increasing evidence demonstrates that EVs have a vital role in mediating intercellular communication via transfer of biological molecules from donor cells to neighbouring or distant recipient cells in both physiological and pathological conditions [1–8].

There are in general three types of EV, exosomes, microvesicles and apoptotic bodies, which are classified according to their biogenesis [8–10]. Originating from endosomal compartments, exosomes are generated by intact cells through intra-luminal budding of multivesicular bodies (MVBs) and are released to the extracellular environment when MVBs move to and fuse with the plasma membrane. Exosomes are relatively homogenous in size, ranging from 30 nm to 150 nm in diameter, and contain marker proteins such as tetraspanins CD9, CD63 and CD81, flotillin, ALG2-interacting protein X (ALIX) and tumour susceptibility gene 101 protein [8,10,11]. By contrast, microvesicles are derived by direct outward budding and fission of the plasma membrane, are characterised by varying sizes between 50 nm and 2000 nm in diameter and display surface markers of the cells from which they originate [10]. Apoptotic bodies come from outward blebbing of the plasma membrane of apoptotic cells, with sizes ranging from 50 nm to 5000 nm in diameter, and are characterised by extensive exposure of phosphatidyl-serine on the surface [10]. Currently, EV studies have mainly focused on exosomes and microvesicles because of their essential roles in modulating cell behaviour and fate.

As natural nanoscale agents, EVs can infiltrate tissues and can even penetrate the blood–brain barrier; thus, they have therapeutic potential as drug delivery vehicles [11–13]. Indeed, increasing numbers of studies have used EVs to deliver anticancer microRNAs (miRNAs) and proteins [14,15]. Several Phase I studies using EVs were completed in the early 21st century and no grade II toxicity or maximal tolerated dose was found, indicating the safety of EV administration [13,16–18].

Although most, if not all, cells release EVs, mesenchymal stromal cell (MSC)-derived EVs (MSC-EVs) may have advantages for drug delivery. Compared with other cell types, MSCs produce many EVs and show sustainable and reproducible EV production [19]. MSC-EVs have good stability in human plasma and during storage at −20°C [20,21], and they have high flexibility for modification *in vitro* and *in vivo* [14,22,23]. In addition, unmodified MSC-EVs have shown encouraging therapeutic effects and are well tolerated in various animal models [8,24–26]. For example, MSC-EVs have been used to alleviate liver fibrosis [25], reduce myocardial infarct size [27] and ameliorate induced allergic airway inflammation in immunocompetent mice [28].

Tumour necrosis factor (TNF)-related apoptosis-inducing ligand (TRAIL) is a promising anticancer protein that binds to its cognate death receptor 4 (DR4) or DR5 on target cells, resulting in apoptosis induction in transformed or cancerous cells but not in normal cells [29,30]. It is safe to deliver the agent for therapeutic interventions, with the ligand exhibiting no detectable cytotoxicity to normal tissues in murine and primate models [31,32] or in humans [33]. The protein in its soluble recombinant form (rTRAIL) has been extensively tested as a cancer therapeutic agent *in vitro* and in clinical trials [31,32,34–38]. However, the therapeutic benefits have been limited [39], possibly due to its poor pharmacokinetics and cancer cell resistance [39]. To overcome these shortcomings, efficient TRAIL delivery is essential. We sought to investigate whether MSC-EVs are a good candidate for this purpose. In this study we therefore sought to express TRAIL on MSC-EVs and tested the efficacy of TRAIL-loaded EVs on cancer-cell killing.

## Methods and materials

### Cell culture

Cell culture reagents were purchased from Invitrogen unless otherwise stated. Eleven cancer cell lines were tested, including 3 lung cancer lines (A549, NCI-H460 and NCI-H727), 4 malignant pleural mesothelioma lines (H2795, H2804, H2810 and H2818), 2 renal cancer lines (RCC10 and HA7-RCC), 1 human breast adenocarcinoma line (MDAMB231; M231) and 1 neuroblastoma line (SHEP-TET). A549 and M231 were obtained from Cancer Research UK. Other cell lines were kind gifts from Dr Ultan McDermott in Wellcome Trust Sanger Institute, Cambridge, UK. NCI-H460 and H2810 were cultured in RPMI-1640 medium with 10% foetal bovine serum (FBS); RCC-10 cells were cultured in DMEM/F-12 with 10% FBS; and all other cell lines were grown in DMEM containing 10% FBS. Well-characterised human adult MSCs (passage 1) were purchased from Texas A&M Health Science Centre. TRAIL-transduced MSCs (MSCTRAIL cells) were previously established by transduction of MSCs with lentiviruses expressing human TRAIL [40]. Both MSCs and MSCTRAIL cells were routinely cultured and maintained in α-MEM medium containing 17% FBS. For the isolation of MSC- and MSCTRAIL-derived EVs, cells were cultured in α-MEM medium containing 10% EV-depleted FBS (Cambridge Bioscience). Primary human lung bronchial epithelial cells (HBECs) were previously established in the laboratory [41] and cultured in DMEM/Ham F-12 with additives following the reported description [42].

### Isolation of EVs

To prepare cell-derived EVs, early passage MSCs and MSCTRAIL cells (not older than passage 5) were first cultured in α-MEM medium containing 17% FBS until cells reached 70–80% confluence; then the medium was changed to α-MEM containing 10% EV-depleted FBS (Cambridge Bioscience) for a further 48 hours. Cell-conditioned medium was then collected and centrifuged for 10 min at 300 × g and then again at 2000 × g at 4°C to remove cells and debris, followed by vacuum filtering the medium through 0.22 μm filters (Merck Millipore) to remove large vesicles, concentrating the medium five times using 100-kDa MWCO centrifugal filtering columns (Merck Millipore, UK) and finally ultracentrifuging for 2 hours at 100,000 × g at 4°C. The obtained EV products were washed twice with 0.22 μm membrane-filtered phosphate-buffered saline (PBS) and finally resuspended in PBS for storage in −80°C until use. Alternatively, the isolated EVs were lysed in EV lysis buffer for quantification using a commercial EV quantification kit (EXOCET96A-1, Cambridge Bioscience, UK); for Western blotting, the isolated EVs were lysed in radio immunoprecipitation assay (RIPA) buffer supplemented with protease inhibitor cocktail (Sigma-Aldrich). EV protein yields were determined by using a bicinchoninic acid (BCA) protein assay following the manufacturer’s instructions.

### Transmission electron microscopy (TEM)

Isolated EVs suspended in PBS were absorbed on formvar/carbon-coated nickel grids for 10 min and unbound EVs were washed away with 0.1× PBS. Grids were then fixed in 1% glutaraldehyde for 10 min and negatively stained with 0.3% uranyl acetate in 1.9% ethylcellulose. Excess fluid was removed and grids were air dried before examination and imaging with a Tecnai T12 electron microscope (FEI, Eindhoven, Netherlands).

### EV labelling and cell uptake assay

EVs were labelled with the green lipid membrane dye PKH67 (Sigma-Aldrich) for cell uptake and visualised or labelled with fluorescent-conjugated antibodies that are specific for proteins expressed in EVs. For PKH67 labelling, 3 µg of EVs were stained with 2 µM PKH67 for 5 min at room temperature and then dialysed in PBS for 24 h to remove free dye using a D-tube dialyser (6–8 kDa) (Novagen). For direct immunofluorescence staining, EVs were first incubated with PBS containing 0.2% bovine serum albumin (BSA), followed by incubation with a fluorescein isothiocyanate (FITC)-conjugated mouse anti-human CD63 antibody (MCA2142F, AbD Serotec, UK), or with isotype control antibodies for 1 h at 4°C. Subsequently, the labelled EVs were washed with PBS and precipitated with a commercial EV precipitation solution (ExoQuick-TC, Cambridge Biosciences, UK) to remove unbound antibodies. EVs labelled by FITC-conjugated anti-CD63 antibody were analysed for CD63 expression by flow cytometry; those stained with PKH67 were applied to M231, H2795, A549 and H2810 cells for cellular uptake experiments. In brief, recipient cells were cultured in normal culture medium in wells of chamber slides (Nunc Lab-Tek) and PKH67-labelled EVs or -control PBS were added to wells at 3 µg/ml and incubated for 1 h at 37°C in a 5% CO_2_ incubator. Medium was then removed and cells were washed three times with PBS. Afterwards, cells were fixed for 10 min using 4% paraformaldehyde, washed, mounted with the ProLong Gold Antifade Reagent with DAPI (Invitrogen, UK) and analysed using confocal microscopy (Leica TCS SP2 microscope). For uptake quantification, after washing, cells were subject to fluorescence-activated cell sorting (FACS) analyses.

### Flow cytometry

Flow cytometry analysis was performed to examine protein expression on the EV surface membrane. For the detection of CD63 expression, EVs were labelled with the FITC-conjugated mouse anti-human CD63 antibody (MCA2142F, AbD Serotec, UK), or with a FITC-conjugated mouse isotype IgG (MCA928F, AbD Serotec, UK) and gated as previously described [43]. Alternatively, the isolated EVs were first purified for CD63^+^ vesicles by binding to magnetic Dynabeads coated with a human CD63 antibody (Cat:10606D, Invitrogen), then labelled with Alexa Fluor 647 (AF647)-conjugated antibodies or PE-conjugated antibodies and analysed for TRAIL and tetraspanin (CD63, CD9 and CD81) expression by flow cytometry. Antibodies used include AF647-conjugated mouse anti-human CD63 (H5C6, BD Pharmingen), AF647-conjugated mouse IgG1 k isotype control (MOPC-21, BD Pharmingen), PE-conjugated mouse anti-human CD9 (Cat:555372, BD Pharmingen), PE-conjugated mouse anti-human CD81 (Cat:555676, BD Pharmingen), PE-conjugated mouse anti-human CD63 (Cat:557305, BD Pharmingen) and PE-conjugated mouse IgG1 k isotype control (Cat:559320, BD Pharmingen). PE-conjugated anti-human DR4 antibody (Cat: 564180, BD Pharmingen), PE mouse anti-DR5 (Cat: 565499, BD Pharmingen) and PE mouse IgG1 isotype (Cat: 559320, BD Pharmingen) were used to label cellular surface DR4 and DR5. FACS median fluorescence intensity (MFI) of labelled cells was used to quantitate EV or cellular surface protein expression levels or measure cell uptake of EVs labelled by green lipid membrane dye PKH67.

### Western blot analysis

MSCTRAIL cells or EVs were lysed in RIPA buffer for total protein extraction and the protein concentration in the lysate was determined using a BCA protein assay. 30 µg of cellular lysate proteins or 20 µg of EV proteins for each sample were resolved on 4–12% polyacrylamide sodium dodecyl sulphate gels and analysed by means of immunoblotting with primary rabbit anti-human TRAIL (c-terminal) (ab42121, Abcam) antibody and primary mouse anti-CD9, anti-CD81 and anti-CD63 antibodies (Invitrogen, UK). An HRP-conjugated secondary antibody against rabbit or mouse IgG (Cell Signaling Technology, UK) was used accordingly. Signals were detected with the Amersham ECL plus western blot detection system (GE Healthcare).

### Cell treatment and apoptosis analysis

Cancer cells or HBECs were plated at 8000 cells per well in 96-well plates and cultured for overnight, then were treated with agents for 24 h to examine agent cytotoxicity. The tested agents include MSC-EVs, MSCTRAIL-EVs (MSCT-EVs), recombinant TRAIL (rTRAIL; amino acids 114–281, Peprotech), the pan-caspase inhibitor Z-VAD-FMK (1 mg/ml, Sigma), a neutralising monoclonal anti-TRAIL antibody (10 ng/ml, Sigma, Cat. No. T3067), a CDK9 selective inhibitor SNS032 (S1145, Selleckchem, UK) (300 nmol/l) and control medium, which were added to culture medium in combination or alone. After treatment, both floating and adherent cells were collected and stained with AF647-conjugated Annexin V (Invitrogen) and 2 μg/ml DAPI (Sigma), followed by cell death assessment by means of flow cytometry. Annexin V+/DAPI− cells were considered to have undergone early apoptosis, Annexin V+/DAPI+ staining considered as late apoptosis, Annexin V−/DAPI+ cells considered to be dead not by apoptosis and both Annexin V and DAPI negative cells were regarded as viable. In parallel, cell apoptosis was also assessed using the Annexin V-PE/7-AAD cell apoptosis assay kit (Cat: 559763, BD-Pharmingen) following the manufacturer’s instructions.

### Active caspase-8 staining

Cancer cells were treated for 24 h with rTRAIL or EVs to induce apoptosis. The treated cells were then harvested and stained with the active caspase-8 inhibitor Red-IETD-FMK that was conjugated to sulfo-rhodamine (K198-25, Bio-Vision) according to the manufacturer’s instructions. Subsequently, the stained cells were analysed for active caspase-8 expression by means of flow cytometry.

### Statistical analysis

Data were analysed with the use of GraphPad Prism 6 software (GraphPad Software), one-way ANOVA followed by Dunnett’s post-test or with Student’s t-test. Significant probability values are denoted as * *p* < 0.05, ** *p* < 0.01 and *** *p* < 0.001.

## Results

### Isolation and characterisation of MSC-derived EVs

We have previously established TRAIL-overexpressing MSCs (MSCTRAIL cells) that express both membranous and secreted TRAIL [40,44–46]. It has been shown that TRAIL can be an EV cargo released into the extracellular milieu by some types of cells under certain conditions [4,43,47]. We therefore hypothesise that MSCTRAIL cells mass-produce and secrete functional TRAIL-loaded EVs.

**Figure 1.**
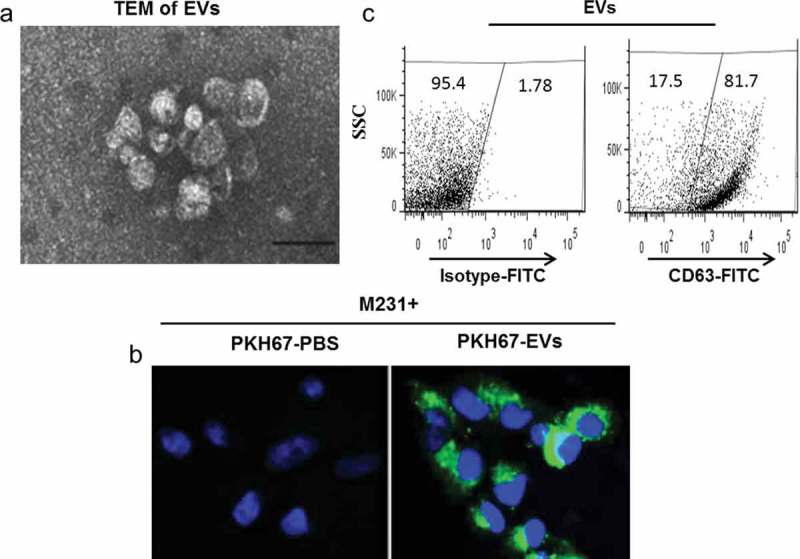
Isolation and characterisation of MSC-derived extracellular vesicles (MSC-EVs). (a) Negative contrast micrograph of MSC-EVs examined and imaged by transmission electron microscopy (TEM), scale bar 100 nm. (b) EVs were labelled by the lipid membrane dye PKH67 (green) and taken up by MDAMB231 (M231) cells, followed by confocal microscopy examination; nuclei of M231 cells were labelled with DAPI (blue). (c) Flow cytometry analysis of EVs. EVs were labelled with a FITC-conjugated antibody against CD63 and compared with those labelled with a control FITC-isotype antibody. Top, a representative dot plot of at least three experiments showing the percentage of CD63-expressing EVs among the tested sample; bottom, quantitation of median fluorescent intensity (MFI) of labelled EVs.

**Figure 2.**
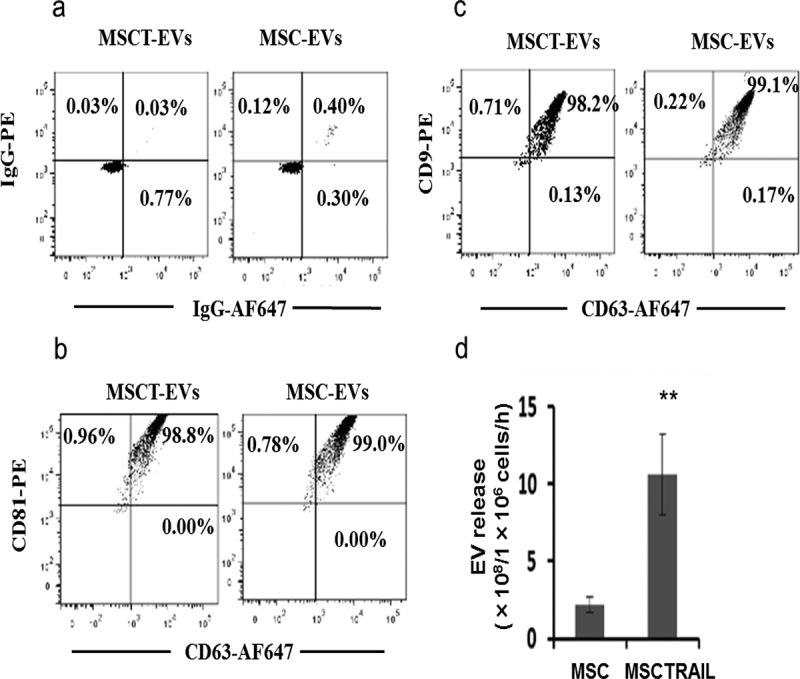
Flow cytometry analysis of tetraspanin CD63, CD9 and CD81 expression on purified EVs, which were captured and purified with magnetic latex beads coated with mAb against CD63 and labelled with PE-conjugated antibodies; the labelling was done in detergent-free buffer aiming for EV surface protein labelling only. (a) Purified EV-bead complexes were stained with IgG-PE and IgG-AF647 isotype antibodies. (b) EV-bead complexes were labelled with CD63-AF647 and CD81-PE antibodies. (c) EV-bead complexes were labelled with CD63-AF647 and CD9-PE antibodies. (d) EV quantification showed that MSCTRAIL cells released more EVs than parental MSCs and empty vector lentivirus-transfected cells (MSCEV). EV number was determined by measuring the EV-enriched acetyl-CoA acetylcholinesterase (AChE) activity with a commercial EV quantification kit, values are mean ± SEM, *n* = 4; ** *p* < 0.01, by Student’s t-test.

To validate this hypothesis we first isolated and characterised MSC-derived EVs. EVs were isolated from medium conditioned by MSCs or by MSCTRAIL cells by sequential ultracentrifugation combined with 0.22 μm ultrafiltration and were examined with transmission electron microscopy. As shown in Figure 1(a) the obtained product appears as membrane-enclosed vesicles of approximately 50–70 nm in diameter, of similar morphology to that previously observed [48,49]. The isolated EVs were subsequently labelled with the lipid membrane dye PKH67, were taken up by MDAMB231 (M231) cells and were visualised using fluorescence microscopy (Figure 1(b)). The prepared EVs were also labelled with a FITC-conjugated anti-CD63 antibody and were analysed by flow cytometry. The isolated vesicles were more than 80% CD63-positive, indicating that exosomes were mainly extracted by this preparation route (Figure 1(c)). These CD63^+^ EVs were purified following binding to magnetic beads coated with monoclonal CD63 antibody. The purified MSC- and MSCTRAIL-derived vesicle-bead complexes were labelled with PE- or AF647-conjugated isotype antibodies (Figure 2(a)), or antibodies against CD9, CD63 or CD81 and were shown to be CD63-, CD9- and CD81-positive by flow cytometry (Figure 2(b,c)), further confirming their exosomal property. Interestingly, CD81 showed the highest expression level on isolated vesicles, CD63 in the middle and CD9 expressed the lowest levels of expression on isolated vesicles (see supplementary Figure 1 online). Finally, EV production was measured for MSCs, MSCs transduced with empty lentiviruses (MSCEV) and MSCTRAIL cells using a commercial kit. Interestingly, results showed that TRAIL-expressing MSCs release significantly more EVs (10.61 ± 2.62 × 10^8^ EVs per h per 1 × 10^6^ cells) than both MSCs (2.21 ± 0.52 × 10^8^) and MSCEVs (2.29 ± 0.47 × 10^8^) whilst MSCs and MSCEV cells showed similar EV release (Figure 2(d)).

### MSCTRAIL-derived EVs express membranal TRAIL

**Figure 3.**
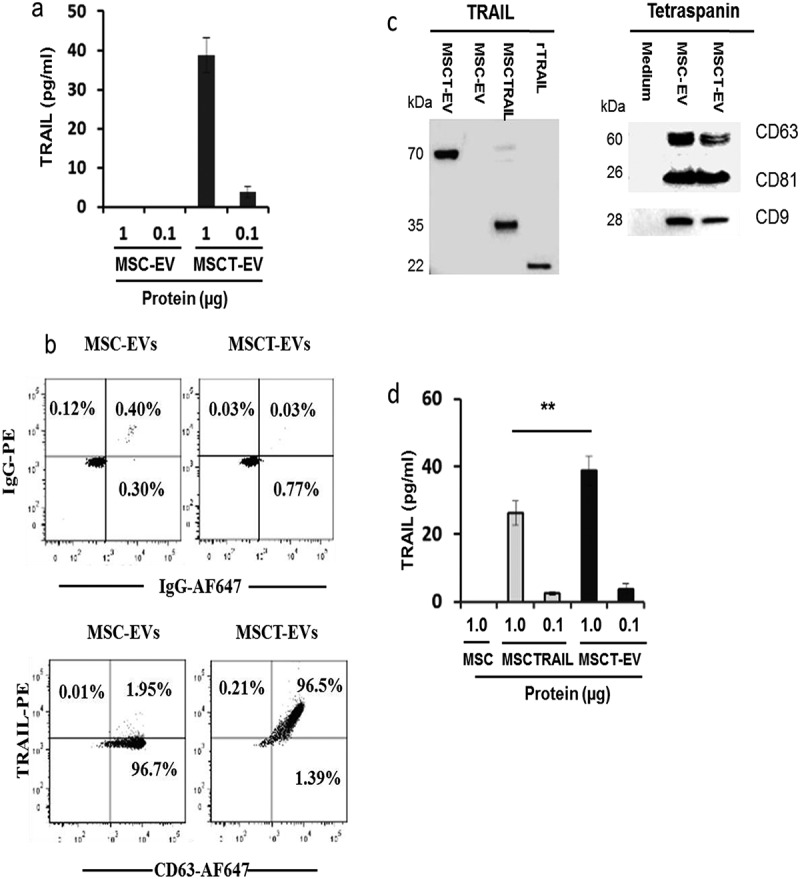
Detection of TRAIL and tetraspanin expression in isolated MSC-EVs. (a) Measurement of TRAIL expression in EVs with a commercial ELISA kit. (b) Flow cytometry analyses of TRAIL and CD63 expression in purified EVs. MSC- and MSCT-EVs were purified with latex beads coated with CD63 antibody and labelled with IgG-PE, IgG-AF647 or TRAIL-PE and CD63-AF647 antibodies, respectively. (c) Western blotting detection of TRAIL and tetraspanin CD63, CD9 and CD81 in EVs and MSCTRAIL lysates; for each sample 30 µg of cellular proteins or 20 µg of EV proteins were analysed; EV preparation of medium without cell culture and 1 ng of rTRAIL (amino acids 114–281, PeproTech, USA) were used as negative and positive controls, respectively. (d) Comparison of TRAIL expression levels in MSCs, MSCTRAILs and MSCT-EVs using a commercial TRAIL-specific ELISA kit. Values are mean ± SEM, *n* = 4; ** *p* < 0.01.

We next examined TRAIL expression in prepared MSCTRAIL-derived EVs using a highly specific commercial ELISA. The results showed that 38.8 ± 4.4 pg of TRAIL was expressed in 1 μg of MSCTRAIL-derived EV proteins (MSCT-EV); by contrast, MSC-derived EVs (MSC-EV) showed no detectable TRAIL expression (Figure 3(a)). MSCT-EVs were purified with beads coated with CD63 antibody and TRAIL expression was analysed by flow cytometry using a PE-conjugated TRAIL-specific antibody. Results confirmed that CD63^+^ MSCT-EVs express TRAIL (96.5% ± 2.8% positive) whilst MSC-EVs were negative for TRAIL expression (1.95% ± 0.5% positive) (Figure 3(b)). TRAIL expression on MSCT-EVs was also examined by western blotting. As shown in Figure 3(c), TRAIL was expressed in MSCT-EVs as a single band of ~70 kDa, which potentially corresponds to a hexameric form of TRAIL [4]. By contrast, the major fraction of TRAIL in the MSCTRAIL cellular lysate was resolved as a band of ~35 kDa and only a minor fraction presented as a 70-kDa band. The tetraspanins CD63, CD81 and CD9 were also detected by western blotting in analysed EVs, confirming that the isolated vesicles were mostly exosomes (Figure 3(c)).

Using a commercial ELISA, TRAIL expression was compared between MSCT-EVs and their originating cells (Figure 3(d)). The results showed significantly higher TRAIL expression in MSCT-EVs (38.8 ± 4.4 pg TRAIL per 1 μg of EV proteins) than in MSCTRAIL cells (26.2 ± 3.53 pg TRAIL per 1 μg of cellular proteins), suggesting the relative enrichment of TRAIL molecules in EVs. Taken together, these data demonstrate that MSCs, when transduced to express TRAIL, can release TRAIL into the extracellular environment via EVs.

### TRAIL delivery by MSCT-EVs induces apoptosis in cancer cells

**Figure 4.**
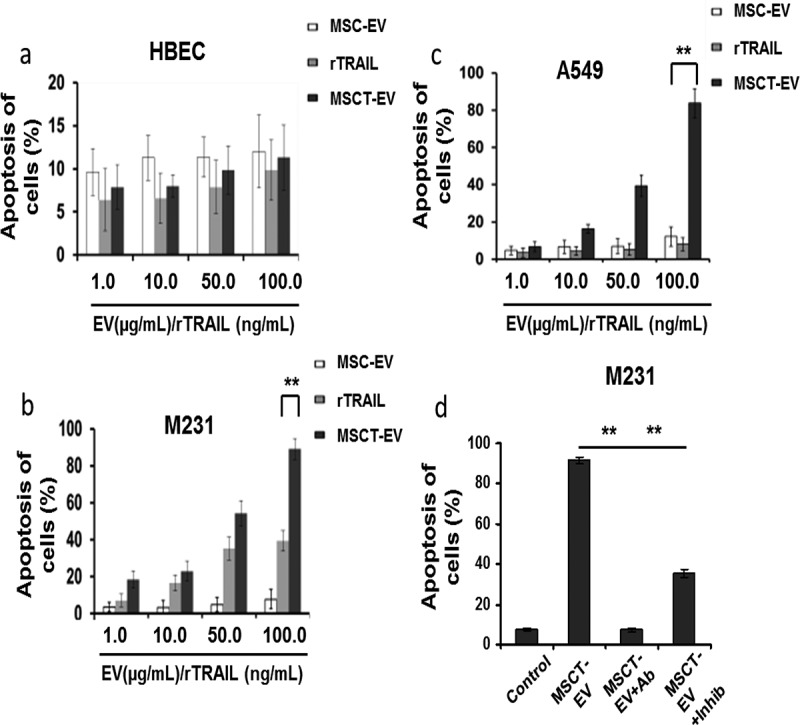
MSCT-EVs induced apoptosis of cancer cells with high efficiency. Cells were cultured in 96-well plate and treated for 24 h with MSC-EVs or MSCT-EVs with concentrations varying from 1.0 to 100.0 µg/ml, or treated with 1.0–100.0 ng/ml rTRAIL, followed by cell harvesting and labelling with Annexin V-AF647 and DAPI for apoptosis assay by flow cytometry. (a) Treatment of primary human bronchial epithelial cells (HBECs). (b) Treatment of M231 cells. (c) Treatment of A549 cells. (d) Treatment of M231 cells with 100 ng/ml of TRAIL-neutralising monoclonal antibody (Ab) (T3067, Sigma-Aldrich) alone, 20 µmol/l of pan-caspase inhibitor Z-VAD-FMK (inhib) alone, or with MSCT-EVs (100 µg/ml) alone, or with MSCT-EVs in combination with Ab (MSCT-EV+Ab) or with inhibitor (MSCT-EV+inhib). Data represent averages ±S.E.M, *n* = 4. * *p* < 0.05, ** *p* < 0.01 compared with MSC-EV control, by one-way ANOVA followed by Dunnett’s post-test.

The isolated EVs were used to treat the breast adenocarcinoma line M231, the lung adenocarcinoma cell line A549 and primary normal human bronchial epithelial cells (HBECs) to test their cytotoxic activity on these cells. No cytotoxic effects were observed on HBECs after MSC-EV, MSCT-EV or rTRAIL treatment (Figure 4(a)). By contrast, rTRAIL and MSCT-EV induced apoptosis in M231 cells in a dose-dependent manner, whilst no cell killing was observed with the MSC-EV control (Figure 4(b)). Interestingly, TRAIL presented by MSCT-EVs is more efficient at cancer cell killing than rTRAIL as 100 µg/ml MSCT-EV that contains 3.88 ng TRAIL/ml induced significantly more apoptosis in M231 cells compared with 100 ng/ml of rTRAIL (Figure 4(b)). As seen in Figure 4(c) and consistent with previous observations [40,50], the A549 cell line is fully resistant to rTRAIL. However, TRAIL delivery by MSCT-EVs induced significant apoptosis in A549 cells in a dose-dependent manner (Figure 4(c)). The A549 killing capacity of MSCT-EVs was confirmed using both Annexin V/DAPI and Annexin V/7-AAD methods, which both showed similar apoptosis-inducing activity of TRAIL-expressing EVs on A549 cells (supplementary Figure 2(a–f)).

The pan-caspase inhibitor Z-VAD-FMK and the TRAIL-neutralising antibody T3067 were tested on M231 cells, either alone or together with MSCT-EVs. Results showed that inhibition of caspase activity or neutralisation of TRAIL significantly reduced MSCT-EV-induced apoptosis in M231 cells whilst inhibitor or antibody only showed no effects (Figure 4(d)), suggesting that the pro-apoptotic activity of MSCT-EVs is caspase dependent and requires the engagement of TRAIL receptors. Taken together, these data demonstrate that TRAIL delivery by MSCT-EVs efficiently induces apoptosis in cancer cells.

### MSCT-EVs overcome TRAIL resistance of cancer cells

**Figure 5.**
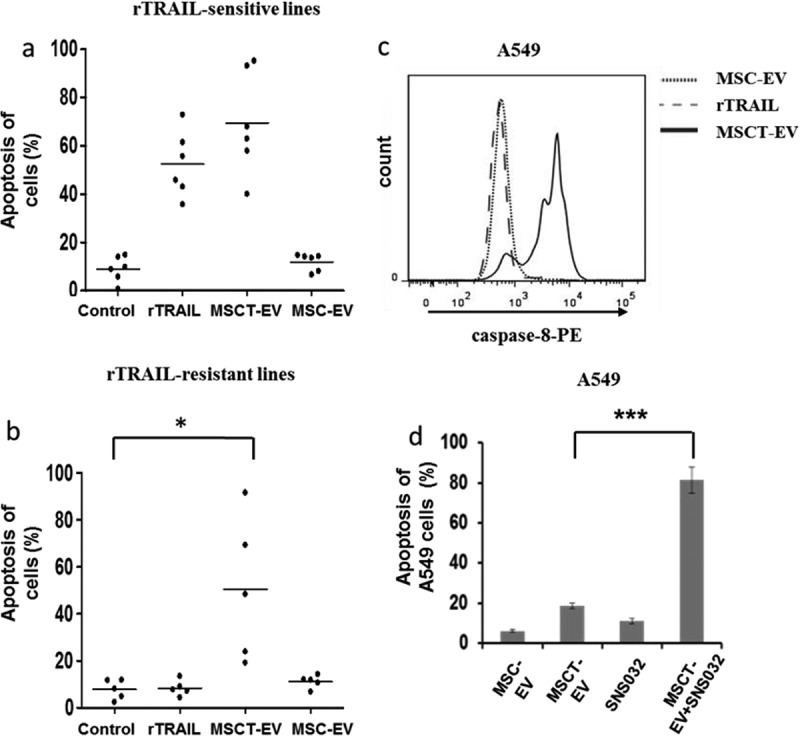
MSCT-EVs efficiently killed rTRAIL-resistant cancer cell lines. (a) Six rTRAIL-sensitive cancer cell lines (H2795, H2804, NCI-H460, NCI-H727, SHEP-TET and M231) were cultured in 96-well plates and treated with MSC-EVs (100 µg/ml), MSCT-EVs (100 µg/ml), rTRAIL (100 ng/ml) or with control medium for 24 h, followed by analysis by cell apoptosis assay. (b) Five rTRAIL-resistant cancer cell lines (H2810, H2818, HA7-RCC, RCC10 and A549) were treated with same agents like in (a) and analysed for cell apoptosis. (c) Caspase-8 activity was examined by flow cytometry in A549 cells, which were treated with MSC-EVs (100 µg/ml proteins), MSCT-EVs (100 µg/ml proteins) or with rTRAIL (100 ng/ml) for 24 h. Treated cells were labelled with the active caspase-8 binding dye Red-IETD-FMK and analysed by flow cytometry. (d) CDK9 inhibition by SNS032 drastically enhanced apoptosis induction in A549 cells by MSCT-EVs. Cell were treated with MSC-EV (10 µg/ml), MSCT-EV (10 µg/ml) or SNS032 (300 nmol/l) alone or with MSCT-EV (10 µg/ml) in combination with SNS032 (300 nmol/l) for 24 h, and analysed by cell apoptosis assay. Data represent averages ± SEM, *n* = 4. * *p* < 0.05, *** *p* < 0.001, analysed by Student’s t-test.

The cytotoxic activity of MSCT-EVs was further examined in a panel of 11 established cancer cell lines, consisting of 3 lung cancer lines (A549, NCI-H460 and NCI-H727), 4 mesothelioma lines (H2795, H2804, H2810 and H2818), 2 renal cancer lines (RCC10 and HA7-RCC), 1 breast cancer line (M231) and 1 neuroblastoma line (SHEP-TET). These cell lines were first treated with rTRAIL (100 ng/ml) and showed varying levels of sensitivity in their response (Figure 5(a,b)). They were grouped accordingly into those that were rTRAIL sensitive (apoptosis ≥35%; 6 cell lines including H2795, SHEP-TET, M231, NCI-H460, NCI-H727 and H2804) or those that were rTRAIL resistant (apoptosis ≤15%; 5 cell lines including A549, H2810, H2818, HA7-RCC and RCC10). These two groups of cell lines were then treated with MSCT-EVs and MSC-EVs (100 µg/ml EV proteins) and analysed for the induction of apoptosis using flow cytometry. In the treatment of TRAIL-sensitive cell lines, MSCT-EVs showed effective apoptosis induction (70% ± 28%), although levels were only slightly higher than those induced by rTRAIL (55% ± 15%). MSC-EVs induced levels of apoptosis that were not different to control. Interestingly, in the treatment of TRAIL-resistant cell lines in which rTRAIL did not induce significant levels of apoptosis, MSCT-EVs were capable of causing apoptotic cell death where MSC-EVs were not although the efficacy greatly varied between different cell lines (Figure 5(b)). Apoptosis induction rates for individual cancer cell lines were shown in supplementary Figure 3(a,b) to distinguish which cell lines responded well or not to treatment. DR4 and DR5 are responsible for the TRAIL signalling pathway. To investigate if cancer cell sensitivity to TRAIL is linked with DR4 and DR5 expression, all the tested cancer cell lines and a normal HBECs were examined for DR4 and DR5 expression using PE-conjugated antibody surface staining and FACS analysis. The results showed that all tested cell lines express DR4 and DR5 but with greatly varying levels (supplementary Figure 4(a,b)). Both sensitive and resistant groups show higher DR5 expression levels than DR4, however no significant differences in DR4 or DR5 expression were revealed between the two groups. Therefore cancer cell sensitivity to TRAIL treatment may be associated with multiple factors, not only with DR4 and DR5 expression levels.

Cell uptake of EVs was quantitated by FACS and compared within two sensitive lines M231 and H2795 and two resistant lines A549 and H2810. The results revealed no significant uptake differences either between rTRAIL-sensitive and resistant cell lines or between MSC-EVs and MSCT-EVs (supplementary Figure 5(a,b)), indicating that the cytotoxicity of MSCT-EVs on TRAIL-resistant cell lines is not due to higher cellular uptake of EVs.

In addition, apoptosis induction was accompanied by activation of caspase-8 in TRAIL-resistant cells treated with MSCT-EVs but not with MSC-EVs or rTRAILs (Figure 5(c), shown for A549 cells), which confirmed the delivery of a pro-apoptotic signal by MSCT-EVs. CDK9 inhibition has been shown to synergise with TRAIL treatment to induce apoptosis in TRAIL-resistant cancer cells [50]. We therefore tested the combinational treatment of MSCT-EVs and a CDK9 inhibitor, SNS032, in TRAIL-resistant A549 cells [51]. As shown in Figure 5(d), while the low concentration of MSCT-EVs (10 µg/ml proteins) alone only showed very limited cancer cell-killing activity (19.3% ± 2.1%), the combination of MSCT-EVs and SNS032 greatly enhanced the level of apoptosis induction (82.5% ± 8.3%).

We have therefore demonstrated that MSCT-EVs can be used as broad-spectrum anticancer agents, which are capable of at least partially overcoming TRAIL-resistance in cancer cells.

## Discussion

In this work we show that TRAIL-transduced MSCs secrete EVs expressing surface TRAIL molecules (MSCT-EVs), that MSCT-EVs are highly efficient at selectively inducing apoptosis in cancer cells and that delivery of TRAIL by MSCT-EVs at least partially overcomes TRAIL resistance in cancer cells.

In this study MSC-EVs were prepared using ultracentrifugation combined with 0.22 μm filtration aiming to preferentially isolate exosomes. The results showed that ˃80% of isolated vesicles were CD63^+^ exosomes expressing TRAIL, suggesting our preparation is mainly composed of exosomes. Although this method removes any microvesicles bigger than 220 nm, it is impossible to separate exosomes from microvesicles of smaller sizes (below 220 nm). However, the preparation can be further purified by sucrose density gradient ultracentrifugation as exosomes have a higher sucrose density (1.13–1.19 g/ml) compared with that of microvesicles (1.04–1.07 g/ml).

Studies from other groups suggest that the source of MSCs from which the EVs are produced could be important in determining whether the MSC-EVs exert anticancer activities or promote tumour growth. For example, human umbilical cord Wharton’s jelly MSC (hWJMSC)-derived EVs have been demonstrated to reduce T24 bladder carcinoma growth [52]. Similarly, EVs isolated from normal human bone marrow (BM)-MSCs were reported to suppress cell cycle progression and to induce apoptosis in liver carcinoma (HepG2) and Kaposi’s cells [53]. However, Roccaro et al. [54] found that BM-MSC-EVs from patients with multiple myeloma could promote multiple myeloma tumour/cell growth, whereas EVs from BM-MSCs isolated from healthy patients inhibited the growth of multiple myeloma tumour/cells both *in vitro* and *in vivo*. So, for the therapeutic production of EVs, it will be important to start with an appropriate source of MSCs to avoid possible adverse effects.

TRAIL secretion via EVs seems to be a natural approach used by certain types of cells to modulate the function and behaviour of target cells at local or at distant sites. For example, human Jurkat and normal T cells, upon activation by phytohemagglutinin (PHA) or by an anti-CD59 antibody, release TRAIL-bearing EVs [55]. Also, human placental syncytiotrophoblasts secrete TRAIL-loaded EVs, which induce apoptosis in activated immune cells [4]. In addition, human colorectal cancer cells were found to secrete TRAIL-expressing exosomes, resulting in the suppression of surrounding immune cells [47]. All of these cells can be potentially used for production of TRAIL-carrying EVs. However, MSC-derived EVs may be the best choice considering their good stability [20,21], high modification flexibility *in vitro* and *in vivo* [14,22,23], inherent therapeutic benefits and high tolerance [24–26].

MSCs do not normally express endogenous TRAIL [40], but upon stimulation with TNF-α, MSCs express membrane-bound TRAIL but not the secreted type [56]. Here we demonstrate that TRAIL-transduced MSCs secrete TRAIL-carrying EVs. Interestingly, TRAIL-transduced MSCs showed enhanced overall EV secretion compared with untransduced MSCs or empty vector lentivirus-transfected MSCs. The underlying enhancing mechanism is not clear but the endoplasmic reticulum (ER) stress-related enhancement of protein secretion via EVs might be involved [57–59].

TRAIL in its soluble recombinant form, i.e. rTRAIL, has been extensively tested for cancer therapy both *in vitro* and *in vivo*. As demonstrated previously [40,44,46] and also in this study, rTRAIL shows a relatively low efficiency for cancer cell killing compared with MSC- or MSC-EV-mediated delivery of TRAIL. The clinical trials that have so far been carried out using rTRAIL had used a high administration dose of up to 30 mg kg^−1^, possibly because of its limited bioavailability and low activity; despite this, the obtained therapeutic benefits were poor [39]. Resistance to this ligand is another reason for its poor clinical performance in cancer treatment [39]. However, the high cancer cell-killing efficiency shown by TRAIL-expressing MSCT-EVs in this study indicates that TRAIL delivery by EVs may improve the clinical performance of TRAIL. It is not clear why EV-displayed TRAIL is more efficient at inducing apoptosis than the recombinant soluble type. We also noticed that TRAIL-expressing MSCs are much more effective in inducing apoptosis than rTRAIL [40,44]. It was recently shown that higher order clustering of TRAIL receptors may be necessary for effective activation of the extrinsic death pathway [60,61]. Indeed, TRAIL oligomerisation has been demonstrated to be necessary for its efficient induction of apoptosis in target cells [62]. TRAIL molecules expressed in MSCT-EVs or by MSCs are localised on the lipid membrane and therefore possibly allow higher order clustering of the ligand to take place as a result of movements permitted by the fluidic nature of the bilayer lipid membrane; this could be the reason why MSCT-EVs induce more apoptosis in target cells than rTRAIL.

In addition to their superior TRAIL-presenting efficiency, as drug delivery vehicles EVs have other attractive advantages. First, EVs are natural nanoparticles that may infiltrate a wide range of tissues following systemic administration. It has been reported that EVs can even cross the brain-blood barrier to deliver their cargoes [14]. Second, there is a high degree of flexibility in how EVs can be modified both *in vitro* and *in vivo*. As demonstrated in this study, MSCs can be genetically modified to produce EVs loaded with therapeutic molecules such as TRAIL. Third, drugs or siRNAs can be directly loaded into prepared EVs [22] or applied to parental cells and incorporated during the biogenesis of EVs [63]. For example, MSCs treated with paclitaxel have been shown to take up the drug and to later secrete paclitaxel-loaded EVs, which are capable of inhibiting human pancreatic adenocarcinoma (CFPAC-1) cell proliferation and of reducing tumour growth by up to 50% [63]. In this study we show that combinational treatment of cancer cells with MSCT-EVs and the CDK9 inhibitor SNS032 drastically enhanced cancer cell-killing efficacy. In the future it would be really interesting to test whether loading SNS032, paclitaxel, TRAIL-sensitising siRNA or other small therapeutic molecules into the prepared TRAIL-expressing MSCT-EVs could have even greater or possibly synergistic anticancer efficacies *in vitro* and *in vivo*.

Finally, EVs have an inherent homing ability allowing them to migrate towards damaged tissues or cancers *in vivo* [64]. For example, EVs, when systemically administered, were found to home to xenograft breast cancer tissues in mice [65]. The underlying mechanism remains unclear although an acidic pH within solid tumours may increase EV uptake [66]. Moreover, EVs can be engineered to increase their tumour tropism. El-Andaloussi et al. [22] have demonstrated that dendritic cell (DC)-derived EVs can be engineered to fuse a neuron-specific RVG peptide to the EV membrane protein LAMP2B, resulting in enhanced brain homing. Similarly, EVs can be modified to display an epidermal growth factor receptor (EGFR)-binding peptide GE11 on their surface for targeted delivery of a therapeutic miRNA to xenograft breast cancer tissues that express EGFR [65]. EGFR is overexpressed or aberrantly activated in many tumours including lung adenocarcinoma [67], suggesting that EGFR could serve as a target in the EV-based cancer drug delivery system. While our prepared TRAIL-bearing EVs are still to be investigated for their tumour-homing properties and their anticancer efficacy *in vivo*, it could be possible to enhance their cancer-targeting capacity by fusing tumour antigen-binding peptides such as GE11 to the EV membrane.

Taken together, results indicate that TRAIL-expressing MSCT-EVs could potentially be developed into an innovative anticancer therapy.
