# Supplementary material for: TRAIL delivery by MSC-derived extracellular vesicles is an effective anticancer therapy
Source: J Extracell Vesicles. 2017 Jan 18;6(1):1265291. doi: 10.1080/20013078.2017.1265291 (PMC5328331; doi:10.1080/20013078.2017.1265291)
Supplement: Supplementary_material__2_.pdf [file ZJEV_A_1265291_SM0414.pdf]

Supplementary Figure 1

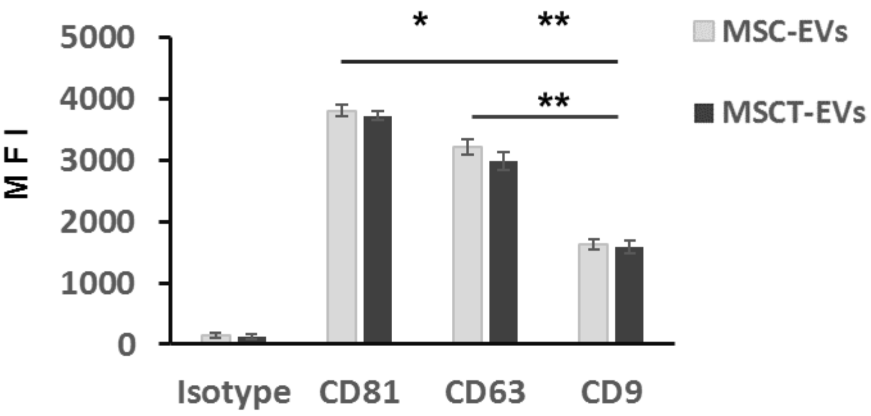

Supplementary Fig.1 Flow cytometry quantification of tetraspanin CD81, CD63 and CD9 expression levels on surface of purified MSC-EVs and MSCT-EVS. EVs were bound to CD63 mAb-coated latex beads and purified, then followed by surface labelling in detergent-free buffer with PE mouse Abs against human IgG (isotype), CD81, CD63 and CD9, respectively, and analysed by FACS. Median PE fluorescence intensity (MFI) of samples was used to assess expression levels of tetraspanin proteins. Data represent averages ±S.E.M, n=4. * $p<0.05$, ** $p<0.01$, by one-way ANOVA test.

Supplementary Figure 2

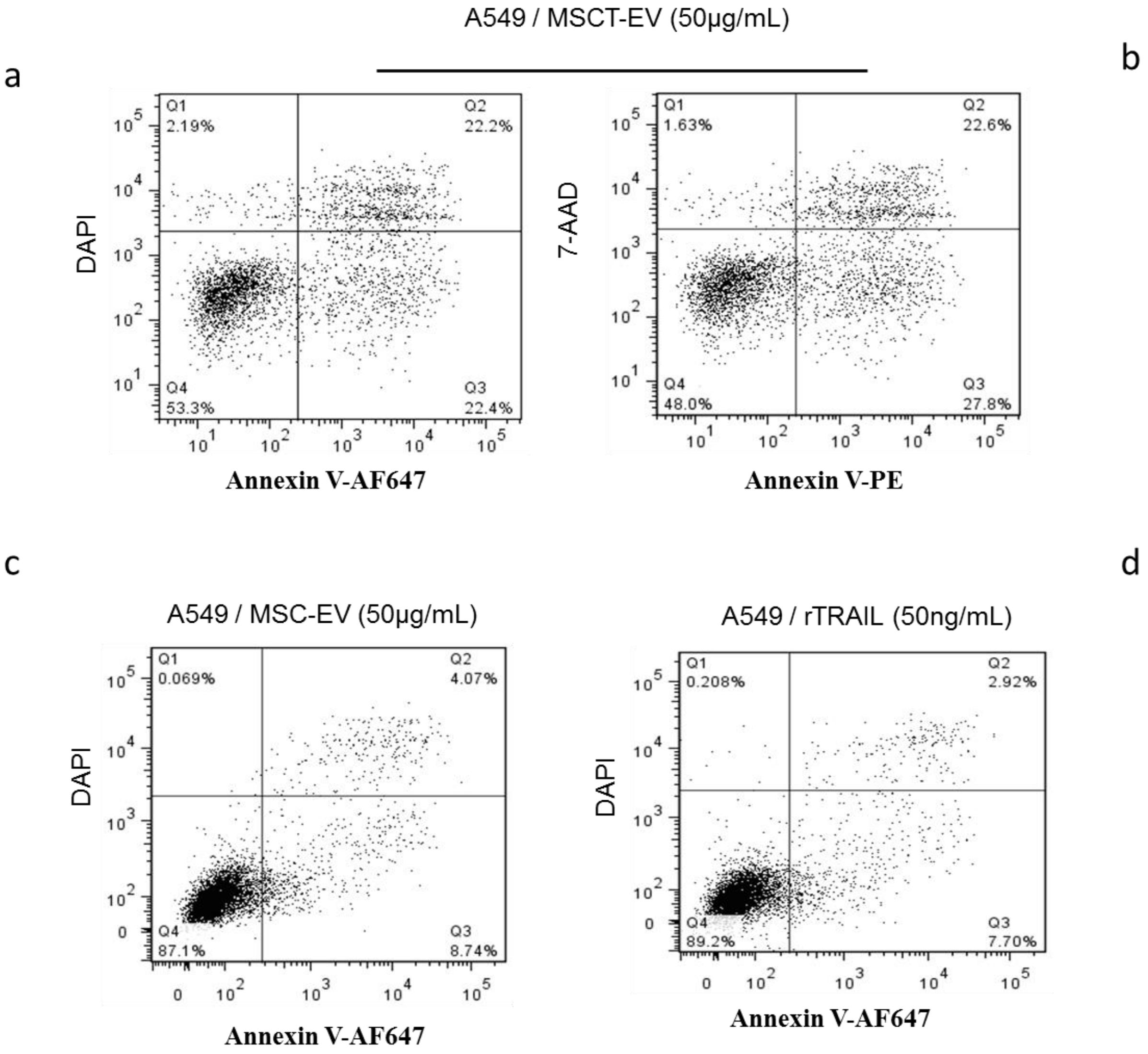

Supplementary Fig. 2 Apoptosis assay with Annexin V/DAPI and Annexin V/7-AAD, no significant differences observed between two methods. A549 cells were treated with MSCT-EVs (50μg/mL), MSC-EVs (50μg/mL), and rTRAIL, respectively, followed by apoptosis assay with Annexin V-AF647/DAPI and Annexin V-PE/7-AAD.（a）FACS dot plot of apoptosis assay by Annexin V/DAPI for MSCT-EV treatment; (b). Apoptosis assay by Annexn V/7-AAD for MSCT-EV treatment; (c). FACS dot plot of apoptosis assay of cells treated by MSC-EV; (d). FACS dot plot of apoptosis assay of cells treated by rTRAIL; (e). Comparison of cellular apoptosis rates measured by Annexin V/DAPI and Annexin V/7-AAD, respectively; (f). Comparison of cellular non-apoptotic death rates measured by Annexin V/DAPI and Annexin V/7-AAD, respectively.

Supplementary Figure 3

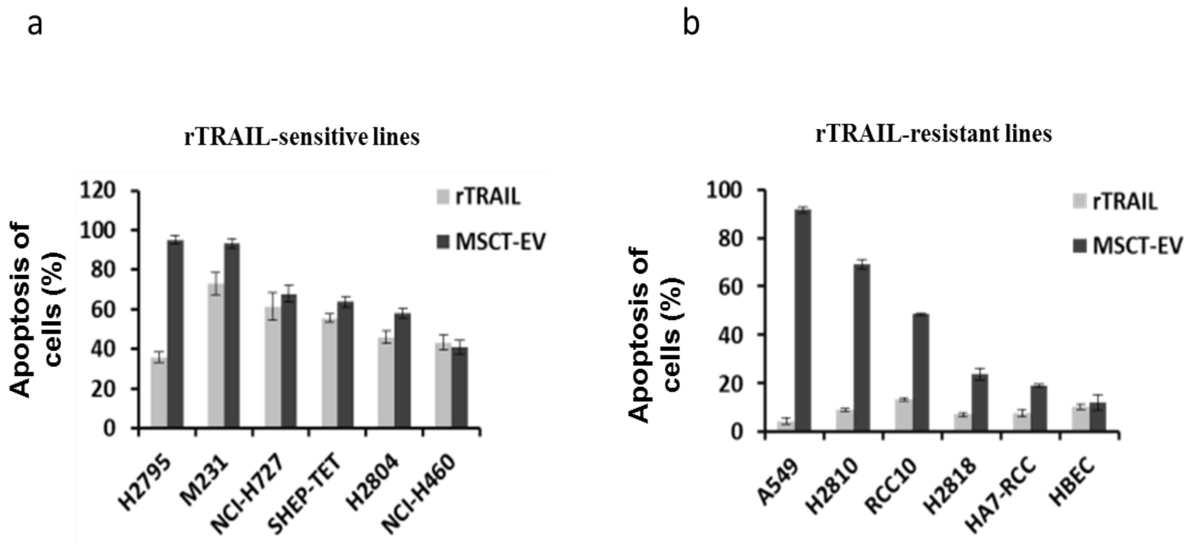

Supplementary Fig. 3 Apoptosis assay of cells treated by rTRAIL or MSCT-EV. (a) Apoptosis rate display of six individual cell lines that are rTRAIL-sensitive; (b). Apoptosis rate display of six individual cell lines that are rTRAIL-resistant.

Supplementary Figure 4

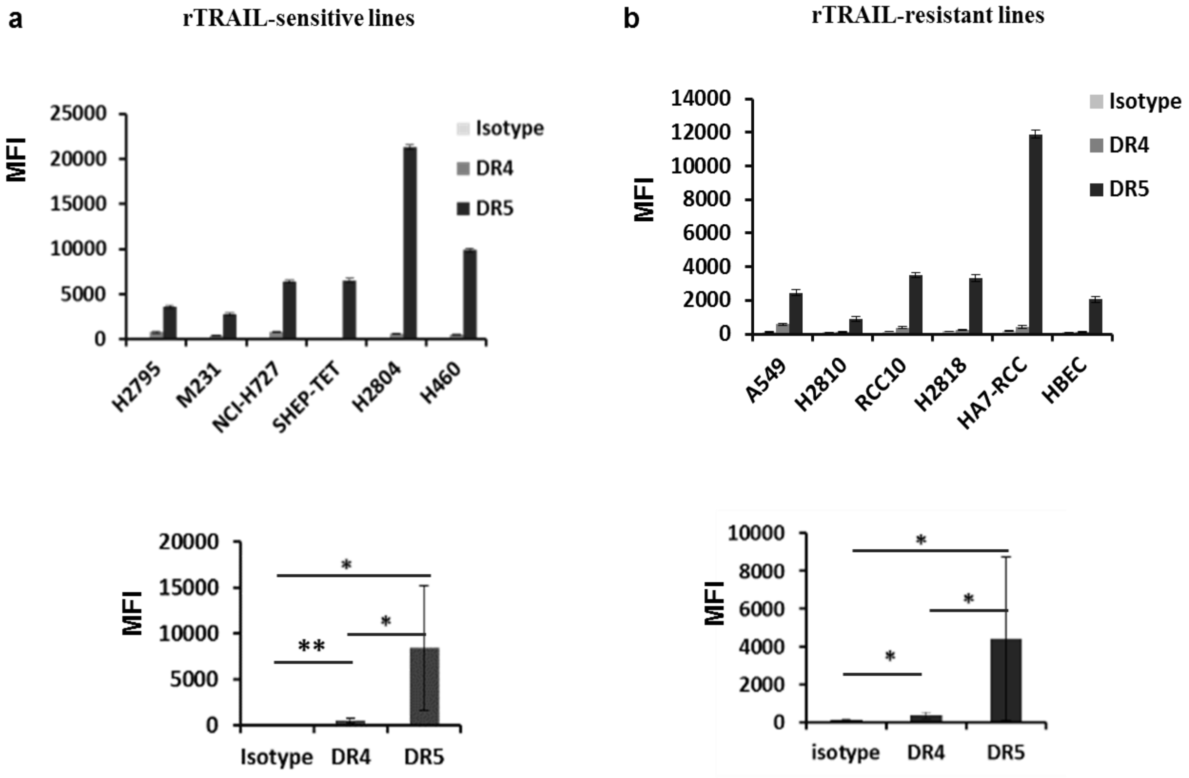

Supplementary Fig. 4 The expression pattern for the death receptors (DR4 and DR5) on the various human cancer cell lines examined for apoptosis and normal human bronchial epithelial cells (HBEC). Cells were labelled in detergent-free buffer with PE mouse anti-human DR4, or anti-DR5 antibody or with PE mouse IgG as isotype control, followed by flow cytometry assay to measure cell surface DR4 and DR5 expression levels. (a). Top, median fluorescence intensity of PE labelling of rTRAIL-sensitive cell lines; bottom, comparison of DR4 and DR5 expression levels. (b). Top, median fluorescence intensity of PE labelling of rTRAIL-resistant cancer cell lines and HBEC ; bottom, comparison of DR4 and DR5 expression levels. * p<0.05, **p<0.01, by one-way ANOVA test.

Supplementary Figure 5

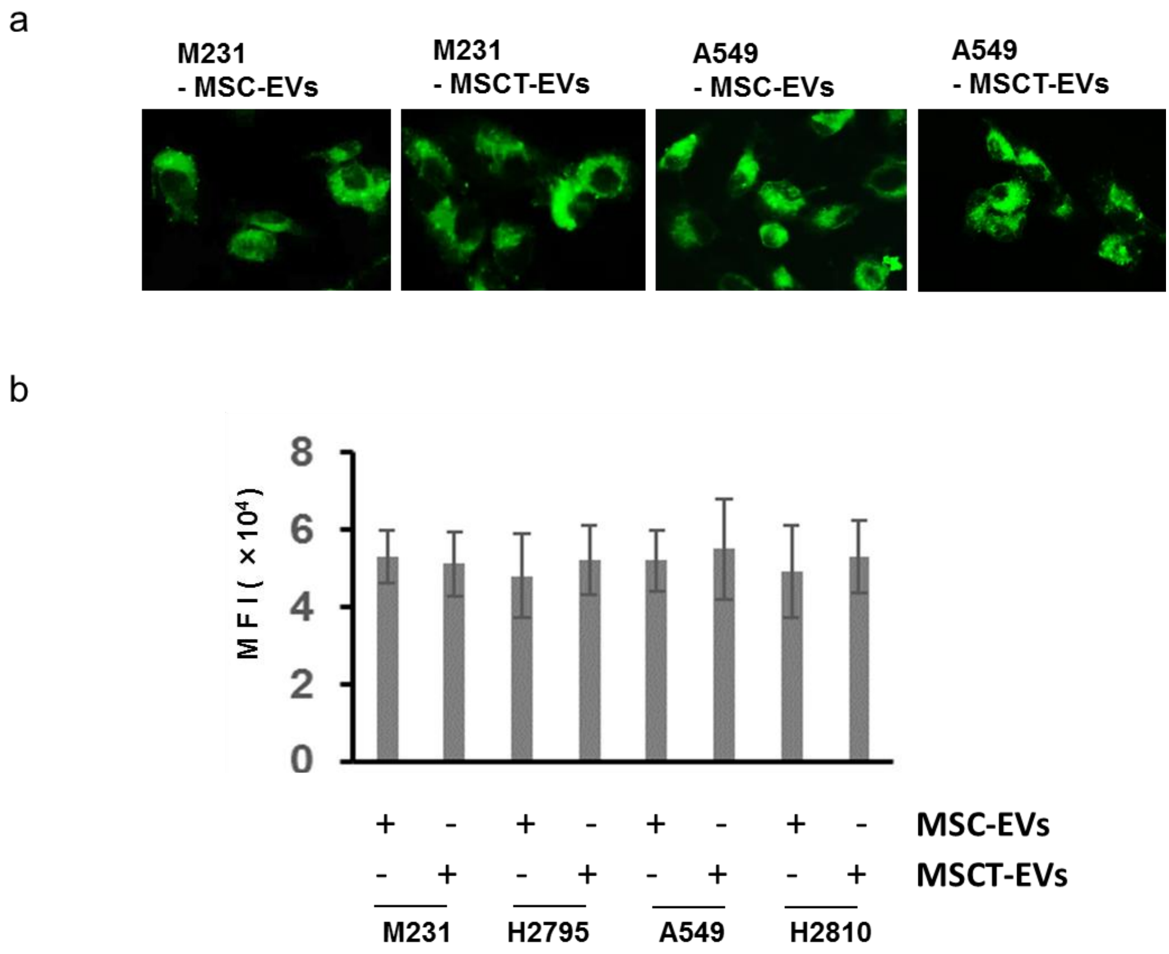

Supplementary Fig. 5 Uptake of EVs was compared within two rTRAIL sensitive lines M231 and H2795 and two resistant lines A549 and H2810; uptake was quantitated by FACS median fluorescence intensity (MFI) of cells, which had taken up EVs labelled with green lipid membrane dye PKH67. (a) Confocal microscopy examination of M231 and A549 cells that had taken up PKH67 labelled MSC-EVs or MSCT-EVs. Magnification 200x ; (b) Flow cytometry quantification of labelled EV uptake by cells. MFI of tested cells is used to assess EV uptake levels. No significant differences were observed among cell lines and between MSC-EVs and MSCT-EVs.
